# Selenium at the **N**eural **B**arriers: **A** **R**eview

**DOI:** 10.3389/fnins.2021.630016

**Published:** 2021-02-05

**Authors:** Nikolay Solovyev, Evgenii Drobyshev, Bastian Blume, Bernhard Michalke

**Affiliations:** ^1^Institute of Technology Sligo, Sligo, Ireland; ^2^Institut für Ernährungswissenschaft, Universität Potsdam, Potsdam, Germany; ^3^Research Unit Analytical BioGeoChemistry, Helmholtz Center Munich – German Research Center for Environmental Health (GmbH), Neuherberg, Germany

**Keywords:** selenium, selenoprotein P, low molecular weight selenium species, blood–cerebrospinal fluid barrier, blood–brain barrier, selenium transport, brain-gut axis, LRP8

## Abstract

Selenium (Se) is known to contribute to several vital physiological functions in mammals: antioxidant defense, fertility, thyroid hormone metabolism, and immune response. Growing evidence indicates the crucial role of Se and Se-containing selenoproteins in the brain and brain function. As for the other essential trace elements, dietary Se needs to reach effective concentrations in the central nervous system (CNS) to exert its functions. To do so, Se-species have to cross the blood–brain barrier (BBB) and/or blood–cerebrospinal fluid barrier (BCB) of the choroid plexus. The main interface between the general circulation of the body and the CNS is the BBB. Endothelial cells of brain capillaries forming the so-called tight junctions are the primary anatomic units of the BBB, mainly responsible for barrier function. The current review focuses on Se transport to the brain, primarily including selenoprotein P/low-density lipoprotein receptor-related protein 8 (LRP8, also known as apolipoprotein E receptor-2) dependent pathway, and supplementary transport routes of Se into the brain via low molecular weight Se-species. Additionally, the potential role of Se and selenoproteins in the BBB, BCB, and neurovascular unit (NVU) is discussed. Finally, the perspectives regarding investigating the role of Se and selenoproteins in the gut-brain axis are outlined.

## Introduction

The crucial role of the essential trace element selenium (Se) for the brain was already reported in the study of Weber et al. (1991) demonstrating the alleviation of intractable seizures in children with a low level of glutathione peroxidase (GPX) activity following Se supplementation. A commonly accepted Se metabolism concept includes the transformation of dietary Se to hydrogen selenide (HSe^–^), which serves as an intermediate between reductive metabolism of Se and excretory pathways, i.e., water-soluble methylated Se compounds (Chatterjee et al., 2003; Ogra and Anan, 2009) and selenosugars (Juresa et al., 2006; Kuehnelt et al., 2006; Rayman et al., 2008). Importantly, hydrogen selenide and its activated form selenophosphate and other reactive low molecular mass (LMM) chemical species of Se are thought to be relevant to the majority of Se biological activity being metabolic precursors of selenoproteins (Loef et al., 2011; Weekley and Harris, 2013). For a detailed description regarding Se absorption and metabolism, the reader is referred to the specialized reviews, e.g., Combs et al. (2013), Roman et al. (2014), Cardoso et al. (2015), and Vindry et al. (2018).

Se is known to contribute to several crucial physiological functions in mammals: antioxidant defense, fertility, thyroid hormone metabolism, and immune response (Hadaszadeh and Beggs, 2006; Rayman, 2012; Schomburg, 2012, 2017; Solovyev et al., 2019). Biological functions of Se in humans (Rayman et al., 2008; Rayman, 2012) manifest themselves primarily via 25 selenoproteins (Savaskan et al., 2003; Arner, 2010; Zhang et al., 2010), highly specialized proteins that have the 21^st^ proteinogenic amino acid selenocysteine (Sec) at their active center (Kryukov et al., 2003; Arner, 2010; Weekley et al., 2011). Hitherto, the functions of a couple of selenoenzymes are rather well described. First of all, antioxidant selenoenzymes are often in the spotlight, including GPXs types I-IV and VI (GPX1-4, 6), thioredoxin reductases type I-III (TXNRD1-3), and methionine sulfoxide reductase B (MrsB) (Arner and Holmgren, 2000; Davis et al., 2012; Rayman, 2012; Brigelius-Flohe and Maiorino, 2013; Kim, 2013). Another relatively well-studied group of selenoprotein are iodothyronine deiodinases type I-III (DIO1-3), which are involved in thyroid hormone metabolism (Köhrle et al., 2000). Other selenoproteins are somewhat less studied, yet their functions seem quite diverse (Papp et al., 2007). For instance, selenoproteins S (SELENOS), N (SELENON), M (SELENOM), T (SELENOT), and F (SELENOF, previously known as 15 kDa selenoprotein (Gladyshev et al., 2016) are endoplasmic reticulum-associated proteins, involved in the unfolded protein response (Bar-Nun, 2005; Ye et al., 2005) and, potentially, other less explored functions.

An important feature of Se metabolism and selenoprotein expression is a highly hierarchic structure. This hierarchy refers to the protein species, organs, and body compartments, with the brain ranging atop of all other organs and tissues. Brain and cerebrospinal fluid (CSF) levels of Se are independent on blood Se level (Tondo et al., 2010; Solovyev et al., 2013); so the brain is protected from Se deficiency (Zhang et al., 2008). The other hierarchic aspect is connected with the patterns of expression of certain potentially more essential selenoproteins (Novoselov et al., 2005) in certain tissues, first of all in the brain, to maintain these important selenoproteins at a high level even at Se deficiency. Conversely, the production of other selenoproteins is severely deprived under Se shortage (Savaskan et al., 2007; Reeves and Hoffmann, 2009; Zhang et al., 2019). Such a Se-utilization hierarchy amongst selenoproteins and body compartments is related to the sophisticated regulation of selenoprotein expression (Papp et al., 2007; Kim et al., 2011). Within the selenoprotein transcription hierarchy, Dio1 holds the top position. The main Se transporting protein – SELENOP – is found in an intermediary position on this selenoprotein transcription ranking. The various forms of GPXs show a scattered picture: GPX2 and GPX4 are less affected by Se deficiency than GPX1 and GPX3 (Sunde, 2012).

Recently, the introduction of genomic, autoradiographic, and proteomic techniques (Guo et al., 2018), as well as advances in chemical speciation (Michalke et al., 2018; Sargent et al., 2019), opened new insights in the studies of brain Se biochemistry and neurotoxicology (Schweizer et al., 2004; Solovyev, 2015). For further details concerning the role of Se in human health (Köhrle et al., 2000; Rayman, 2012; Steinbrenner and Brigelius-Flohé, 2015), the brain and brain disease (Schweizer et al., 2004; Pillai et al., 2014; Cardoso et al., 2015; Solovyev, 2015; Solovyev et al., 2018), metabolism (Steinbrenner and Sies, 2013; Weekley and Harris, 2013; Vinceti et al., 2016), and nutrition (Navarro-Alarcon and Cabrera-Vique, 2008; Torres-Vega et al., 2012) the reader is referred to the specialized reviews.

Se is an essential trace element for the human body and specifically for the human brain (Ingold et al., 2018), but it also can be highly neurotoxic depending on intake and speciation (Rayman, 2012; Vinceti et al., 2014; Solovyev, 2015; Michalke et al., 2018). The nutritional requirement for Se was first demonstrated in 1957 (Schwarz and Foltz, 1957), which was underpinned by the discovery of Se-dependent GPXs (Rotruck et al., 1973). Nevertheless, the optimal dietary intake of Se induced intensive debates for a long time, which are still going on (Sunde, 2006; Vinceti et al., 2013a, 2017b; Roman et al., 2014). Currently, the values of ca. 20–70 (Gammelgaard et al., 2008; Schomburg, 2012; Vinceti et al., 2013a; Weekley and Harris, 2013) or 40–50 μg Se per day are most commonly cited in the literature as an optimum Se intake (Sunde, 2006; Combs et al., 2013). Tolerable upper intake level was set by the Institute of Medicine of the National Academy of Sciences of the United States as 400 μg per day for adults (Boyd, 2011). European Food Safety Authority (EFSA) set adequate Se intake as 70 μg/day for adults and 85 μg/day for lactating women (EFSA Panel on Dietetic Products Nutrition and Allergies (NDA), 2014). For the rodents, the generally recommended levels of Se in the chow are ca. 0.04–0.10 μg Se/g diet (Yang et al., 1989; Sunde et al., 2005; Sunde and Raines, 2011), which may correspond to ca. 1–2 μg Se daily in rats. The exact optimal intake of Se in rodents seems to be dependent on exact breed, age, and Se speciation.

Notably, the neuroprotective role of Se compounds is not exhausted with antioxidant effects of Se species, but also appeared to have a role in *de novo* selenoprotein synthesis, regulation of calcium channels, and mitochondrial biogenesis (Uguz and Naziroglu, 2012). Remarkably both Se-deficient and Se-excessive diet in mice lead to an increased level of iron in the hippocampus; however, in the cerebral cortex, only Se-deficient diet led to iron accumulation (Sharma et al., 2019). Increased iron in brain tissue causes reactive oxygen species (ROS) formation via Fenton reaction, inducing ferroptosis and finally leading to neurodegeneration (Kim et al., 2015; Stockwell et al., 2017; Cobley et al., 2018). This indicates that Se metabolism may cross-effects the regulation of other metal levels and can lead to a wide range of consequences with pathological effects.

Importantly, as for any other nutritional compounds, dietary Se needs to reach effective concentrations in the CNS to exert its vital function (Campos-Bedolla et al., 2014). To do so, Se-species have to cross the blood–brain barrier (BBB) and/or blood–cerebrospinal fluid (CSF) barrier (BCB). Crossing the barriers as well as subsequent promoting antioxidant activity appear to be, to some degree, dependent on the chemical form, since the organic form of Se was proven to be more powerful in increasing the expression and activity of TXNRD, GPX1 and GPX4 (Song et al., 2014). TXNRD plays an important role in maintaining the redox balance and has protective role inside dopaminergic cells, which are prone to oxidative stress, e.g., under parkinsonian degeneration (Lopert et al., 2012).

Blood–brain barrier and BCB are “guarding systems” of the brain formed mainly by endothelial cells, which separate the central nervous system (CNS) from the general circulatory system of the body, protecting the brain from toxic metabolites and pathogens (Zenaro et al., 2017). BBB and BCB provide trophic support, absorbing nutrients such as amino acids, polyunsaturated fatty acids, and essential trace elements that are vital for brain function (de Wilde et al., 2017). The main interface between the general circulation of the body and the CNS-compartment is the BBB. Endothelial cells of brain capillaries are the primary anatomic units of the BBB, mainly responsible for the barrier function (Abbott et al., 2010). However, brain endothelial cells actively interacting with other brain cells, including neurons, astrocytes, myocytes, pericytes, and extracellular matrix components (Muoio et al., 2014). All these cell types, including BBB endothelial cells, are involved in the regulation of blood circulation, including vasodilation and vasoconstriction, together being referred to as neurovascular unit (NVU).

A peculiar fact on neurodegenerative disorders is that they are normally characterized by an increased ROS production (Loef et al., 2011) and the decline of BBB and BCB (Balusu et al., 2016). For instance, animal (Sengillo et al., 2013) and human studies (Halliday et al., 2016; Skillbäck et al., 2017) indicate the vulnerability of the NVU in Alzheimer’s disease, the most common neurodegenerative disease (Muoio et al., 2014), and both protective and trophic functions of the neural barrier seem to be impaired (Balusu et al., 2016; Zenaro et al., 2017).

Upon entering the body through diet, Se is mainly taken by the liver (Burk and Hill, 2015) to be distributed to the extrahepatic tissue. For the details on Se absorption and general metabolism in the body, the reader is referred to the specialized publications (Ogra and Anan, 2009; Burk and Hill, 2015; Shini et al., 2015; Solovyev et al., 2018; Ha et al., 2019). The current review focuses on Se transport to the brain, including, first of all, selenoprotein P/low-density lipoprotein receptor-related protein 8 (LRP8, also known as apolipoprotein E receptor-2) dependent pathway, and supplementary transport routes of Se into the brain via low molecular weight Se-species. Additionally, a potential role of Se and selenoproteins in the BBB, BCB, and NVU is discussed. Finally, the perspectives regarding investigating the role of Se and selenoproteins in the gut-brain axis are outlined.

## Blood–Brain Barrier, Blood–Cerebrospinal Fluid Barrier, and Neurovascular Unit

The mammalian brain is separated from the general circulation system by the BBB, which is localized in the brain capillaries and pia-subarachnoid membranes, and the BCB localized in the *choroid plexus* of the brain ventricles. The primary contribution to the barrier function belongs to the BBB since at the level of brain micro-vessel endothelium BBB is the major site of blood-CNS exchange (Abbott et al., 2010). BBB plays a crucial role in the maintenance of CNS homeostasis (Erickson and Banks, 2013). The functions of the BBB and BCB include: protection of the brain from pathogens and toxic metabolites, the separation of the brain and periphery neurotransmitter pools, intake of essential nutrients and discharge of metabolites, and maintaining the immune privilege of the brain, where the immune activity is mainly accomplished by internal microglia rather than, e.g., bone marrow or thymus-derived immune cells (Galea et al., 2007; Abbott et al., 2010; de Wilde et al., 2017; Zenaro et al., 2017).

These barriers are physically represented by the so-called tight junctions between brain endothelial cells and epithelial cells, attributed to the special proteins such as occludin, claudins, and the associated proteins zona occludens (ZO-1, ZO-2, and ZO-3), which are highly expressed in brain endothelium (Chen et al., 2009; Steinemann et al., 2016). Another aspect of barrier function is related to the functioning of multiple active transporters, which carry nutrients and metabolites in both directions (Campos-Bedolla et al., 2014; Blanchette and Daneman, 2015). Tight junctions produce high transendothelial cell electrical resistance, impeding ions and small charged molecules from crossing the BBB (Blanchette and Daneman, 2015). Tight junctions also support transporter function by limiting lateral diffusion of membrane proteins (Abbott et al., 2010).

A second interface between the CNS and periphery, formed by the epithelial cells of the *choroid plexus* facing the CSF, the CSF *per se*, and the highly permeable ependyma in the brain ventricles, constitute the BCB (Abbott et al., 2010; Spector et al., 2015). The choroid epithelial interface of the BCB acts together with the BBB, maintaining neuron wellbeing (Johanson et al., 2011). The CSF is an excretion of the *choroid plexus* into the brain ventricular system (Brown et al., 2004) and it is in permanent close contact with the brain in the extraparenchymal cave (Aguilar et al., 1998). The blood comes close to the CSF in two main areas of the brain: over the subarachnoid space in the arachnoid membrane blanket and in the *choroid plexus* of the brain ventricles (Johanson et al., 2011). CSF is bathing and sheathing the brain, protecting it from mechanical stress and contributing to brain homeostasis through constant exchange with brain interstitial fluid (Abbott et al., 2010). This fact predestines CSF to be *that* sample type from living subjects to analyze CNS-related exposure, transport efficiency across neural barriers or metabolic changes in the brain due to neurodegenerative conditions (Solovyev et al., 2013). This holds true as well for Se and selenoproteins or other Se-species.

Barrier functions develop prenatally and are well-formed by birth (Goasdoué et al., 2017). Endothelial progenitor cells invade the neural tissue from the surrounding perineural vascular plexus and enter into the neuroepithelium; neural progenitor cells generate molecular signals driving the migration of the endothelial cells, which in turn secrete cues to recruit pericytes; for details see a review by Blanchette and Daneman (2015). Neural barriers are a highly dynamic system, responding to different signals, including local changes and requirements, and able to be regulated via a number of mechanisms and cell types, in both physiological and pathological conditions (Abbott et al., 2010).

Blood–brain barrier and BCB are sophisticated systems for a direct study in a living organism. Therefore, active attempts are being undertaken to design *in vitro* models of these systems. Such artificial systems could facilitate the investigation of processes across the BBB and BCB. As such models are designed to reproduce and predict the processes across the real barriers. The reliable models must correspond to a relevant set of parameters in the real brain. However, there is still a lack of *in vivo* understanding of many processes at neural barriers, making robust validation of model systems to be associated with noticeable difficulties.

The developed models can be divided into several main types: transwell systems (Helms et al., 2016; Stone et al., 2019), cell aggregate-based models (Urich et al., 2013; Cho et al., 2017), and dynamic systems (Campisi et al., 2018; Jeong et al., 2018; Ahn et al., 2020). In the simplest version, transwell models represent endothelial cells cultured on a matrix-coated permeable membrane inserts for the standard cell culture plates, which divide the cultivation well into two parts, imitating the blood-facing and brain-facing compartments of the barrier. Additionally, astrocytes, pericytes, and neurons can be co-cultured together with endothelial cells to mimic the real vascular environment in the brain more closely (Stone et al., 2019). The advantages of such systems are the simplicity of implementation, low costs, and the possibility to assess the transendothelial electrical resistance (TEER) rather easily as a parameter characterizing modeled barrier integrity. Additionally, such systems are well suited for the screening of permeability coefficients (Wolff et al., 2015); predominantly, in the case of compounds with a passive diffusion mechanism (Garberg et al., 2005). The same transwell membranes can be applied for the modeling BCB (Schroten et al., 2012; Drobyshev et al., 2021). Nevertheless, there is a lack of the relevant cell lines to model the whole sophisticated cell interaction for both BBB and BCB, which is especially problematic for the latter since it is combined with a more model-challenging barrier geometry (Strazielle and Ghersi-Egea, 2011). Overall, non-presentation of some cell types in such models, the absence of blood flow, and a lack of metabolic and neurochemical coupling between the neuronal cells and the barrier components limit the implication of these models (Bagchi et al., 2019).

Dynamic BBB models were designed to overcome the disadvantages of the transwell models associated with the lack of shear stress and close contact of endothelial cells with neuroglia. In these models, endothelial cells and astrocytes are cultured on the inner and outer surface of the porous hollow fibers (He et al., 2014). The culture medium is circulated through the system to achieve shear stress equivalent to that in the physiological conditions. Also, a gas-permeable tubing system is used to keep the O_2_/CO_2_ balance. However, the dynamic BBB model has a lot of shortcomings: it is not possible to visualize the endothelial cells; these models require much higher cell numbers to build-up a tight monolayer and longer cultivation times to reach stable TEER values (Cucullo et al., 2002, 2011). Nevertheless, as these models allow controlling the medium flow, dynamic BBB models were successfully applied for the investigation of the ischemia-induced injury (Cucullo et al., 2008) and antiepileptic drugs (Cucullo et al., 2007). The introduction of microfluidic devices was the next step in the development of dynamic BBB models (Wolff et al., 2015). Due to the miniaturization of the flow chambers and the limitations of the membranes, the conventional dynamic BBB models were mostly discontinued. At the same time, the small size of the flow chambers limits their application for modeling shear stress. However, the active development of the microfluidic BBB systems in recent years demonstrates the potential of these models for a variety of research tasks (Adriani et al., 2017; Jeong et al., 2018; Bhalerao et al., 2020).

Cell aggregate models or “spheroid” models consist of endothelial cells, astrocytes, and pericytes, which are able to self-organize into spherical structures with astrocyte core, surrounded by pericytes and covered with endothelial cells (Urich et al., 2013). Such systems may become a viable alternative to the transwell or microfluidic models for certain implications. The main advantage of these systems is a direct contact between the barrier cells (Gastfriend et al., 2018). Accordingly, the disadvantage of these models is the absence of a simple way to assess barrier function such as TEER measurement and complicated permeability screening (Cho et al., 2017). At the moment, such systems seem to be the most suitable for studying the effects of various compounds on the constitutional cells of the barrier (Nzou et al., 2018; Leite et al., 2019), rather than directly on the barrier functions.

There is a large set of requirements for barrier models: strong barrier function, the presence of a wide range of transporters and receptors, regulation of immune cell trafficking, mimicking a complex interaction of several types of cells, as well as, a dynamic balance between the cells. That makes the implementation of the *in vitro* BBB or BCB models extremely difficult. However, a deeper understanding of the complex nature of BBB and BCB together with the development of the new models and the improvement of the current barrier-modeling techniques indicates that they may become a very useful research tool for studying BBB and BCB in the future. This may include the research on the nutrient transport to the brain tissue and barrier dynamics, including modeling of the NVU functionality.

The concept of NVU was introduced as a structure formed by neurons, astrocytes, basal lamina covered with smooth muscle cells and pericytes, endothelial cells (components of the BBB), and extracellular matrix (Harder et al., 2002). This cellular complex detects the neuronal supply and triggers necessary responses, vasodilation or vasoconstriction, via their anatomical and chemical relationship (Muoio et al., 2014). Importantly, brain endothelial cells are known to gain their specialized BBB functions through interactions with other cells of NVU such as pericytes, astrocytes, and neurons (Canfield et al., 2019), which is crucial for the development, regulation, maintenance of the neural barriers (Daneman et al., 2010a, b).

The decline of BBB and BCB are involved in many neurological diseases (Blanchette and Daneman, 2015), including, e.g., Alzheimer’s (Erickson and Banks, 2013; Zenaro et al., 2017) and Parkinson’s disease (Gray and Woulfe, 2015), epilepsy (Oby and Janigro, 2006), etc. In this respect, BBB is currently drawing more interest if compared to BCB. To conclude, neural barriers, first of all, BBB and other aspects of NVU is a dynamically developing branch of brain research and they may be expected to gain recognition as valid therapeutic targets in the future (Campos-Bedolla et al., 2014).

## Selenium Transport to the Brain – Selenoprotein P and Low Molecular Weight Selenium-Species

Se is an essential trace element necessary for adequate brain function (Cardoso et al., 2015; Solovyev, 2015); however, its uptake by the neuronal tissue should be strictly regulated to prevent toxicity (Burk and Hill, 2009, 2015). Currently, the role of disturbed trace element homeostasis and metal exposure in the brain is being studied intensively. The loss of barrier integrity promotes increased brain exposure to circulating metabolites, inorganic ions, and circulation proteins, which in healthy conditions either cannot enter the brain completely or in a strictly controlled manner only. Both metal ions and leaked proteins (Linert and Kozlowski, 2012; Choi et al., 2017) may modulate amyloidgenesis and other pathological processes in the brain after a “ticketless” transfer through the barrier. Increased brain exposure to mineral elements, present as low-molecular-weight species, bypassing the deteriorating neural barrier may contribute to the general pathologic processes in the brain.

The initial understanding of brain Se transport came from the use of ^75^Se-radioactive tracer experiments (Burk et al., 1991, 2003; Hoppe et al., 2008; Kuhbacher et al., 2009). The presence of ^75^Se in the brain after the injection of labeled ^75^Se-selenite to Se deficient rats was observed only after the appearance of ^75^Se-Selenop in the blood plasma, differentiating the brain from other tissues (Burk et al., 2003). Furthermore, the injection of ^75^Se-labeled Selenop caused five-time higher accumulation of ^75^Se in the Se-depleted rat brain 2 h later than that in Se-sufficient animals (Burk et al., 1991). For more details regarding the early studies on body Se transport, the reader is referred to the review by Chen and Berry (2003).

In recent years, our understanding of Se transport to the brain improved considerably. The central role in Se transport is attributed to SELENOP, a sole selenoprotein in mammals and other vertebrates, containing multiple Se atoms as Sec residues (Kryukov et al., 2003). The biosynthesis of SELENOP, involving the incorporation of multiple Sec moieties, is modulated by two SECIS elements in the 3′ UTR region of SELENOP mRNA, reviewed by Shetty and Copeland (2018a). High high-energy demand from the cell for the incorporation of 7-17 or more, up to 35 (Shetty and Copeland, 2018b), Sec residues, depending on the biological species (Labunskyy et al., 2014), indicates the importance of the protein for the body. SELENOP contains two histidine-rich stretches in the N-terminal domain, which may bind to heparin (Hondal et al., 2001). This differs from the majority of heparin-binding proteins, which bind through basic amino acid sequences containing primarily lysine and arginine (Hileman et al., 1998), but is common for histidine-proline-rich glycoprotein (HPRG) (Burch et al., 1987). Additionally, SELENOP contains a separate heparin-binding site in the N-terminal domain (Burk and Hill, 2009, 2015). Recent studies also showed possible detoxification role of SELENOP, which results from the binding affinity for transition metals such as mercury (Liu et al., 2018). Although SELENOP seems to be a multifunctional protein (Schweizer et al., 2016; Brigelius-Flohe and Flohe, 2017; Solovyev, 2020), body Se transport seems to be its most crucial role (Lobanov et al., 2009). SELENOP is a secreted heparin-binding glycoprotein (Yang et al., 2000), containing ten Se atoms in humans (Chen and Berry, 2003; Rayman, 2012). Circulating SELENOP is mainly produced by the liver (Burk and Hill, 2009; Pillai et al., 2014; Short et al., 2018); however, intracellular expression of SELENOP was reported for neurons (Scharpf et al., 2007), astrocytes (Yang et al., 2000; Steinbrenner et al., 2006), testicular Leydig cells (Koga et al., 1998), adipocytes (Zhang and Chen, 2011), and β-cells of the pancreas (Steinbrenner et al., 2013), at least *in vitro*. Full-length SELENOP and shorter truncated isoforms are detected in the circulation, the latter corresponding both to termination of SELENOP translation at one of the Sec UGA codons (Ma et al., 2002; Kurokawa et al., 2014a) and the action of the proteases (Saito et al., 2004; Kurokawa et al., 2014a, b). SELENOP considerably contributes to the maintenance of body Se homeostasis, mainly orchestrated by the liver (Steinbrenner and Sies, 2009; Solovyev, 2020). The liver directs Se toward essential selenoproteins biosynthesis or excretion (Papp et al., 2007).

As was already mentioned, the human body maintains a specific Se hierarchy (Steinbrenner and Brigelius-Flohé, 2015). The brain ranks high in this hierarchy, being able to maintain relatively high selenoprotein expression under Se deficiency (Burk and Hill, 2009; Solovyev, 2015; Solovyev et al., 2018); together with regulation of selenoprotein expression, SELENOP-dependent Se uptake to the brain seems to play an important role in maintaining this strict hierarchy. In the brain, SELENOP is primarily expressed in astrocytes, but neurons have also been identified as a source of endogenous SELENOP through the entire brain (Steinbrenner et al., 2006; Scharpf et al., 2007), with particularly elevated expression in the putamen and *substantia nigra* (Bellinger et al., 2012). The regulation of SELENOP synthesis seems to be even more sophisticated than that for other selenoproteins, due to the necessity to incorporate multiple Sec elements (Shetty and Copeland, 2018b). As a Se transport protein, SELENOP significantly contributes to Se-dependent brain pathways, including: redox signaling, protein folding, neurochemical signal transduction, and cytoskeleton assembly (Loef et al., 2011; Cardoso et al., 2015).

**FIGURE 1:**
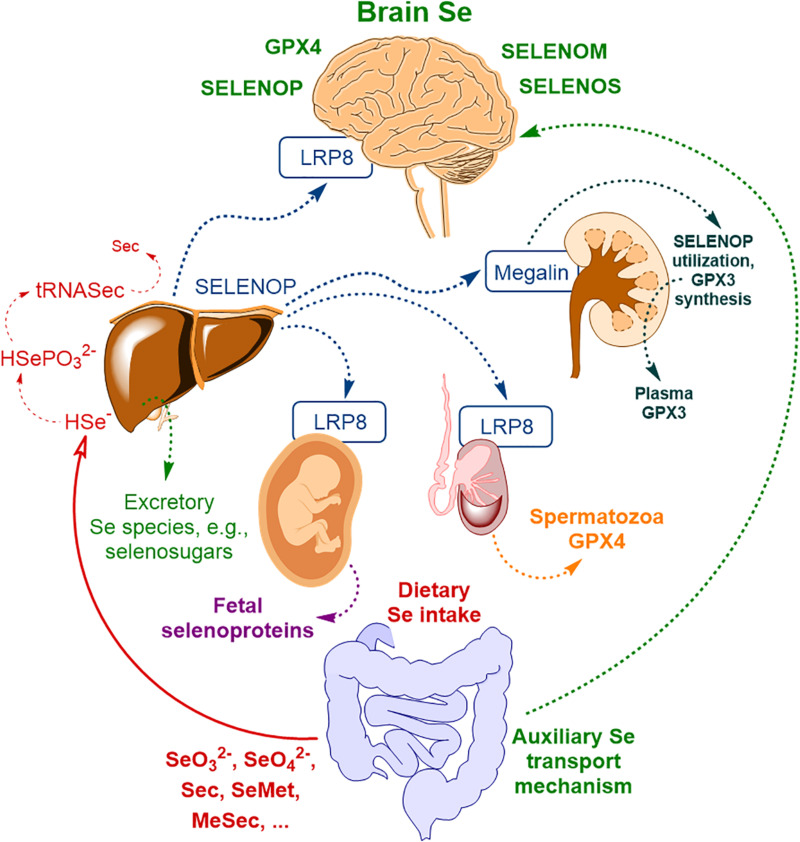
The scheme of body Se homeostasis. Abbreviations: LRP8 – low-density lipoprotein receptor-related protein 8 (LRP8, also known as apolipoprotein E receptor-2, ApoER2), GPX3 – glutathione peroxidase type III, GPX4 – glutathione peroxidase type IV, Sec – selenocysteine, MeSec – methyl selenocysteine, SELENOM – selenoprotein M, SELENOP – selenoprotein P, SELENOS – selenoprotein S; * – auxiliary brain Se transport mechanism, independent of SELENOP, possibly related to selenosugars (Burk and Hill, 2015) and other low molecular weight Se-species (Solovyev et al., 2013) and possibly other minor contributors (please, see text for more detail). Based on Solovyev et al. (2018) with modification.

The majority of the extrahepatic tissues depend on receptor-mediated uptake of SELENOP to maintain adequate selenoprotein expression. Figure 1 illustrates body Se homeostasis and Se transport, based on several sources (Burk and Hill, 2009, 2015; Ogra and Anan, 2009; Solovyev et al., 2013). First of all, the brain, testes, placenta, and kidney rely on receptor-mediated endocytosis of SELENOP. Se delivery to neurons by SELENOP is accomplished via its receptor, low-density lipoprotein receptor-related protein 8 (LRP8, also known as ApoER2, Figure 1) (Burk et al., 2007). SELENOP enters the brain from blood plasma by docking with LRP8 at the BBB in brain capillary endothelial cells (BCECs) and *choroid plexus* epithelial cells (Burk et al., 2014). In other body compartments, LRP8, or another membrane receptor – megalin (also known as LRP2) – is used for SELENOP uptake (Olson et al., 2007, 2008; Chiu-Ugalde et al., 2010; Kurokawa et al., 2012, 2014b); SELENOP *per se* was reported to be in oxidized form for the uptake to take place (Shetty et al., 2018).

This primary mechanism of brain Se uptake was postulated based on mice transgenic studies (first of all, using *Selenop*^–/–^ mice) and is relatively well explored by now. Genetic ablation of SELENOP or LRP8 results in diminished brain Se levels (Hill et al., 2003; Schomburg et al., 2003; Burk and Hill, 2009; Burk et al., 2014) and severe neurological dysfunction upon administration of a Se-deficient diet (Hill et al., 2004; Valentine et al., 2008). The study of *m*RNA levels of selenoprotein and selenoproteom-related genes in *Selenop*^–/–^ mice indicated a considerable reduction of brain selenoprotein expression compared to wild-type mice (Hoffmann et al., 2007). Specifically, the selenoproteins with relatively high expressions in the brain – *Gpx4*, *Selenom*, and *Selenok –* were significantly affected, whereas for selenoprotein W [*Selenow*, an antioxidant selenoprotein with not yet fully understood functions (Whanger, 2009; Yao et al., 2013, 2016)] the expression became nearly undetectable (Hoffmann et al., 2007). Another SELENOP uptake receptor, megalin may also contribute to Se transport to the brain. Megalin is mainly responsible for Se uptake by the kidney (Figure 1) and prevents the discharge of SELENOP in the urine (Olson et al., 2008; Kurokawa et al., 2014a, b). Megalin was demonstrated to be present in the *choroid plexus* of the BCB (Carro et al., 2005; Dietrich et al., 2008); however, its exact contribution to brain Se transport was not systematically studied and it seems to be rather limited since *megalin*^–/–^ mice do not exhibit neurological phenotype associated with Se deficiency (Kurokawa et al., 2014b), typical for *Selenop*^–/–^ or *Lrp8*^–/–^ mice.

**FIGURE 2:**
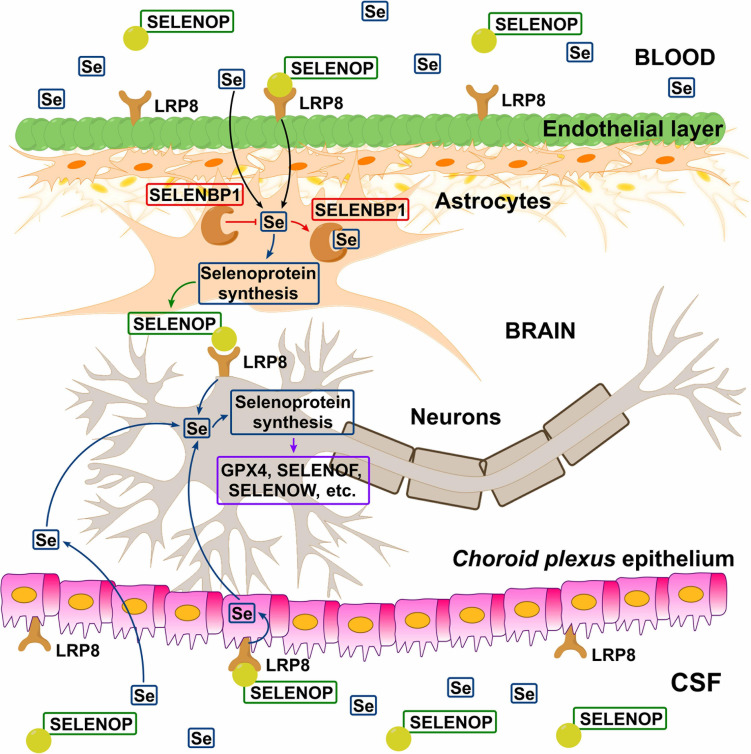
Hypothetical model of Se transport across blood–brain barrier (BBB) and blood–cerebrospinal fluid barrier (BCB). Circulating SELENOP present in blood and CSF is taken up by LRP8-positive cells in the epithelial (BBB) and ependymal (BCB) layers, resynthesized in neighboring astrocytes, and released to supply LRP8-positive neurons with Se. In the astrocytes, SELENBP1 sequesters Se from selenoprotein synthesis and thus negatively regulating SELENOP production. There is also evidence indicating the existence of the SELENOP-independent Se uptake pathway (Figure 1). Reproduced from Sasuclark et al. (2019) with modification.

In a recent study, Sasuclark et al. (2019) explored the cell-type-specific expression of Se-related genes in the mouse and human brain using single-cell RNA sequencing. Transcriptomic data was analyzed in 23,822 mouse and 15,928 human cells for the genes of 22 selenoproteins and 12 other genes, associated with Se-transport and/or metabolism. Different cell types were investigated. High level of expression of LRP8 was observed for brain endothelial cells of the BBB. SELENOP expression considerably overlapped with that in glial fibrillary acidic protein-positive astrocytes and was generally more prominent in white matter. Additionally, SELENOP expression was most robust in the *choroid plexus* and regions lining the brain ventricles (Sasuclark et al., 2019), which is in line with the previous studies (Rueli et al., 2015). Generally, in accordance with the previous findings (Zhang et al., 2008), Sasuclark et al. (2019) indicated that DIO2, SELENOP, and Se-binding protein 1 (SELENBP1) were predominantly expressed in non-neuronal cells (interestingly, SELENOP was co-expressing with SELENBP1 in astrocytes), whereas the vast majority of selenoproteins and Se-related proteins were most abundant in neurons. Importantly, SELENOP expression was maximal in adjacent astrocytes, rather than the ependymal cells directly lining the ventricles. The authors proposed the following model of Se uptake to the brain via SELENOP-LRP8: SELENOP present in blood and CSF is taken up by LRP8-positive cells of BBB and BCB, resynthesized in neighboring astrocytes, and subsequently released to supply LRP8-positive neurons within the brain with Se – Figure 2. SELENOP is known to cross the BCB, being the most abundant selenoprotein and Se-species in the CSF (Solovyev et al., 2013; Mandrioli et al., 2017). For instance, for a collective of 24 neurologically healthy sample donors, a good, age-consistent BBB-integrity value of albumin quotient (*Q*_HSA_) of 5.25 × 10^3^, but higher *Q*-values for GPX (*Q*_GPX_ = 8.31 × 10^3^) and TXNRD (*Q*_TXNRD_ = 21.34 × 10^3^) were observed, demonstrating active transport into the brain-CSF compartment (Solovyev et al., 2013). For SELENOP, even higher CSF-blood quotient (*Q*_SELENOP_ = 91.24 × 10^3^) was reported (Michalke et al., 2017). It may indicate the increased SELENOP transport across the neural barriers, due to high expression of LRP8 at the BBB, which keeps Se levels relatively stable, even during deficiency periods making an adequate Se pool available in the CNS (Zachara et al., 2001). The above-proposed hypothesis is quite intriguing and certainly requires further insight.

Recent investigations by Jin et al. (2020) have shown, that human hepatocellular carcinoma (HepG2) cells secrete SELENOP mainly within exosomes, which are stable against cleavage by plasma kallikrein protease (Saito et al., 2004). Additionally, *in vitro* experiments showed that exosomal SELENOP potentially crossed the BBB and supplemented Se to neuronal cells (mouse neuroblastoma N2a cells), inducing the production of intracellular selenoproteins. Exosomes are a subclass of extracellular vesicles of endosomal origin, which are released from the cells for extracellular communication by the transportation of proteins, DNA or RNA (György et al., 2011). The size of exosomes is ∼40–100 nm in diameter and they are enveloped with a lipid double layer as an outer membrane. The function of exosomes in cell-to-cell communication, protein or RNA transport, immune response regulation, antigen presentation, and non-classical secretion of proteins is reviewed by Simpson et al. (2009). Additionally, Jin et al. (2020) indicated the possible involvement of apolipoprotein E (ApoE) in the regulation of exosomal SELENOP secretion and transport, which probably needs further *in vivo* confirmation. For more detailed information about the cellular uptake of exosomes, the reader is referred to the review of Mathieu et al. (2019). Although further studies on secreted exosomal SELENOP are required, the exosomal transport of selenoprotein through BBB to neuronal cells might be an alternative route for Se delivery into the brain.

The mechanism of SELENOP intracellular turnover is not fully clear. Lysosomal degradation of SELENOP was reported (Kurokawa et al., 2012; Shetty et al., 2018); however, the exact proteolysis pathway requires further insight. Se liberated from SELENOP must then be recycled for the production of new selenoproteins. Selenocysteine β-lyase (Scly), an enzyme that seems to play an important role in Se metabolism, releasing Se atoms from Sec (Seale et al., 2018a; Seale, 2019). The idea that Scly may be responsible for SELENOP’s Se recycling came from the fact that Scly-depleted HeLa cells exhibited a significant decline of selenoprotein production in the case SELENOP was used as a Se source (Kurokawa et al., 2011).

If SELENOP, as a source of Se for selenoprotein synthesis, acts via Scly, it might deliver the highly reactive Sec residues directly to Scly or through an intermediate, in order to decompose Sec and recycle Se (Seale, 2019). It is worth noting that although *Scly*^–/–^ mice have reduced selenoprotein expression, they do not exert any of the *Selenop*^–/–^ phenotypes like male sterility or severe neurologic defects (Raman et al., 2012; Byrns et al., 2014). Byrns et al. (2014) explored the phenotype of double-knockout *Selenop*^–/–^/*Scly*^–/–^ mice, indicating exacerbated neurological phenotype compared to *Selenop*^–/–^ mice, including motor coordination, audiogenic seizures, and brainstem neurodegeneration. *Selenop*^–/–^/*Scly*^–/–^ animals were shown to require supra-physiological Se supplementation to survive (Byrns et al., 2014). Interestingly, the neurological dysfunction related to the inhibition of GABAergic neuron maturation in male double-knockout *Scly*^–/–^/*Selenop*^–/–^ mice could be prevented by prepubescent castration (Pitts et al., 2015). This suggests a competition between the testes and the brain regarding Se-distribution under Se-deficiency or disrupted Se-homeostasis.

The presence of alternative SELENOP-independent transport pathways for Se was identified in the early studies in *Selenop*^–/–^ mice fed Se sufficient diet (Hill et al., 2003; Schomburg et al., 2003). Up-to-now, these pathways are considerably less explored than the main SELENOP/LRP8 pathway, probably, owing to their supplementary function, which may take over only under specific conditions such as SELENOP or LRP8 deficiency. SELENOP-independent Se transport to neuronal tissue may be attributed to selenosugars (Burk and Hill, 2015) and/or other low molecular weight Se-species (Solovyev et al., 2013). Se conversion into methylated species or selenosugars are Se detoxification pathways present in many biological species. However, there is a lack of understanding of the relationship between specific and non-specific Se metabolism (Tobe and Mihara, 2018). Excretory Se-species, such as selenosugars and trimethylselenonium cation (TMSe^+^), were shown to be non-toxic for the astrocytoma and other cell types, compared to selenite (Marschall et al., 2016). These compounds are rather intensively produced under supra-nutritional Se intake (Itoh and Suzuki, 1997; Suzuki et al., 2005; Tsuji et al., 2009), and may contribute to brain Se transport under SELENOP or LRP8 deficiency. In this respect, selenosugars seem to be more feasible candidates, since they might exploit glucose- or other transporters (Campos-Bedolla et al., 2014) to cross the BBB and/or BCB whereas TMSe^+^ is unlikely to be either effectively transported to the brain due to its positive charge and missing capability of effective metabolization in the brain tissue (Suzuki et al., 2006; Jackson et al., 2013). Furthermore, TMSe^+^ seems not to be regularly appearing in human biofluids. Jäger et al. (2016) reported that TMSe^+^ was present among Se-excretory species only for a small fraction of the population. Finally, GPX3 as a secreted isoform of GPX (Steinbrenner and Sies, 2009; Brigelius-Flohe and Maiorino, 2013) may also contribute to supplementary Se transport to the brain. Blood serum GPX3 (Figure 1) is mainly produced by the kidney (Olson et al., 2008) and GPX3 was detected in human CSF (Solovyev et al., 2013; Vinceti et al., 2019), whereas a rather low level of GPX3 expression was reported for the rat *choroid plexus* (Kratzer et al., 2013). Notably, GPX3 contribution to Se transport to the CNS under normal conditions seems to be far lower compared to the SELENOP-associated pathway.

**FIGURE 3:**
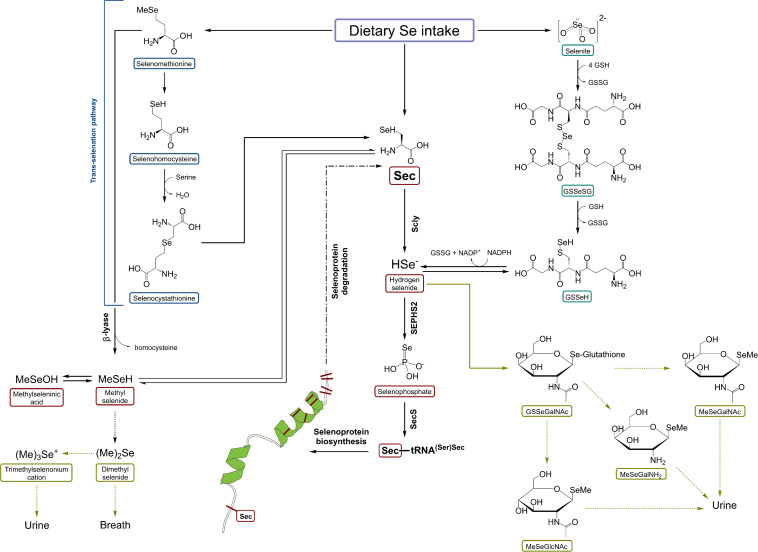
A schematic representation of selenocysteine β-lyase role in Se metabolism. Scly – selenocysteine β-lyase; Sec – selenocysteine; SeMet – selenomethionine; HSe^–^ – hydrogen selenide; GSSeSG – selenodiglutathione; GSSeH – selenoglutathione; SEPHS2 – selenophosphate synthetase 2. Based on Seale (2019) with modification.

Recently, Seale et al. (2018a) presented *in vivo* results on Scly expression and activity under the absence of Sec-rich Selenop, indicating the presence of other pathways of maintaining Sec supply for Scly, which remain to be identified. Dietary Se-species, first of all, SeMet and selenite may substitute SELENOP deficiency. For instance, enhanced reduction of selenite and accelerated trans-selenation pathway for SeMet [analog of trans-sulfuration mechanism, transferring sulfur from methionine to serine to yield cysteine (Jackson et al., 2013)] may be additional sources of Se for selenophospate synthesis and consequently selenoprotein production (Seale, 2019). The activation of these pathways may accelerate the induction of dietary Se-species into selenoprotein synthesis (Esaki et al., 1981; Kumar et al., 1992). Selenite can be reduced to selenide and elemental Se by the action of TXNRD1 (Kumar et al., 1992), an essential selenoprotein ranking high in the selenoprotein hierarchy (Kuhbacher et al., 2009). The scheme of Scly role in Se metabolism is demonstrated in Figure 3 (Seale, 2019). To conclude, the story of Se recycling still poses some unanswered questions; for instance, the exact cellular localization of Scly, whether Scly directly transfers Se to selenophosphate synthetase 2 (SEPH2) or there are other proteins involved, and the exact role of minor intermediates such as selenohomocysteine (trans-selenation pathway) or Se bound to glutathione (selenite reduction pathway) in Se turnover (Seale et al., 2018a; Seale, 2019).

Inorganic Se (selenite and selenate) may cross the BBB and BCB using inorganic anion transporters, which are present in the barriers (Campos-Bedolla et al., 2014). Sulfate transporters [e.g., SLC13: human Na^+^-sulfate/carboxylate co-transporter family (Hu et al., 2020)] may be responsible for carrying selenate across the barrier since it is isomorphic to sulfate. The presence of selenate was observed in human CSF (Vinceti et al., 2013b, 2019; Mandrioli et al., 2017; Violi et al., 2020); however, it is usually mainly attributed to the decaying of CSF selenoproteins, first of all, that of SELENOP (Michalke and Berthele, 2011; Solovyev et al., 2013). Selenite may also employ some inorganic ion transporters but this requires a further warrant. The transport of inorganic Se into the brain appears to be mainly responsible for Se neurotoxicity (Vinceti et al., 2014, 2016), implementing the U-shaped effects of Se on human health (Rayman et al., 2018; Seale et al., 2018b). However, such inorganic Se delivery may become beneficial under severe Se deficiency or malfunction of SELENOP/LRP8 delivery system.

In turn, organic dietary species of Se such as selenoamino acids (SeMet, Se-methylselenocysteine, and to the lesser extent Sec) seem to be capable of entering the brain via aminoacid transporters and, possibly, other routes. Notably, the corresponding mechanistic relationships remain to be elucidated. The key enzyme of the trans-selenation pathway (cystathionine γ-lyase) is known to be expressed in the brain (Diwakar and Ravindranath, 2007; Patel et al., 2018; Seale et al., 2018b). Finally, a minor alternative pathway of Se entering the brain may be related to proteins leaking through the BBB (or BCB), first of all, under pathological conditions, impairing the barrier function, but this notion is rather speculative at the moment. Any general body proteins contain Se as a non-specific substitute for its sulfur analog methionine (Ogra and Anan, 2009). Thus, non-specific leaking of the proteins through the barrier (Pardridge and Mietus, 1980; Lin et al., 2016) may deliver some Se to the brain cells. Particularly, selenized human serum albumin is detected in human CSF (Solovyev et al., 2013; Letsiou et al., 2014). However, the actual contribution of such “backdoor” transport pathways remains elusive.

## Selenium and the Gut-Brain Axis

The human gastrointestinal tract is inhabited by the numerous microorganisms of varied species from different domains of Life, including viruses, archaea, protozoa, bacteria, fungi, and eukaryota (Qin et al., 2010; Welcome, 2019). There is growing evidence of a direct link between gastrointestinal function and the brain (Caracciolo et al., 2014). The gut-brain axis is a bidirectional neurohumoral communication system between the CNS and the enteric nervous system (Collins et al., 2012). For instance, traumatic brain injury activates the gut-brain axis and increases intestinal permeability (Patel et al., 2016); on the other hand, changes of gut microbial composition during neurodevelopment in early life may be detrimental for the CNS and leads to neurological disorders in later life (Louwies et al., 2019). The effect of the gut microbiota on the host’s health is related to the production of biologically active compounds *per se*, competing with the host for essential nutrients, and affecting the host’s immune system (de Vos and de Vos, 2012; Caracciolo et al., 2014; O’Mahony et al., 2015; Yang et al., 2020) and epigenome (Louwies et al., 2019). Intestinal Se absorption depends on the chemical speciation of the element as well as other factors such as the individual’s sex, age, nutritional status, and the composition and activity of the intestinal microbiome (Peters et al., 2018).

The presence of several key selenoproteins including GPXs, SELENOM, SELENOP, and SELENOS as well as SELENBP1 was reported for the intestine, Se status thus affecting gene expression, signaling pathways, and cellular functions in the small and large intestine as well as the gut microbiome composition (Speckmann and Steinbrenner, 2014). Se deficiency is detrimental for the gut barrier function, inducing the disordered intestinal immune response in mice. Additionally, it reduces the levels of neuroactive substances, such as serotonin and melatonin (O’Mahony et al., 2015; Zhai et al., 2019), which are involved in the gut-brain axis (Mawe and Hoffman, 2013; Carabotti et al., 2015). Furthermore, pathological alteration of gastrointestinal flora may lead to diseases, such as inflammatory bowel disease and cancer. The role of Se in these processes remains to be elucidated. The role of Se in the gut disease is outside the scope of the current review and the reader is referred to the specialized publications (Rannem et al., 1998; Speckmann and Steinbrenner, 2014; Kudva et al., 2015; Peters et al., 2018; Kipp, 2020).

The gut microbiota is metabolically highly active and it produces a range of different compounds, including neuroactive molecules, such as acetylcholine, catecholamines, γ-aminobutyric acid, histamine, melatonin, and serotonin. These molecules are essential for regulating peristalsis and sensation in the gut (Petra et al., 2015). Additionally, the presence of gut microbiota considerably affects the uptake and metabolism of the nutrients. Up to 25% of all bacteria have selenoproteins in their genomes (the number varies from 0 to 57) and, thus, they require Se for their growth and metabolism (Kasaikina et al., 2011; Zhang et al., 2019).

In the study of Kipp et al. (2009), male mice were kept on diets for 6 weeks, simulating Se-sufficient (150 μg/kg Se as SeMet) and moderately Se-deficient (86 μg/kg Se) diets in humans. Even this narrow decrease in Se net intake caused the alteration of 952 genes expression – 772 genes were down-regulated and 230 genes were found to be up-regulated. The following pathways were shown to be affected: regulation of protein biosynthesis, response to stress, inflammation, carcinogenesis, and the Wnt pathway (Kipp et al., 2009). In a later study, Kasaikina et al. (2011) studied the composition of gut microbiota in mice kept on Se-deficient, Se-sufficient, and Se-excessive diets. High-throughput sequencing was used for the purpose. They showed gut microbiota to be able to partially sequester dietary Se, limiting its uptake by the host. The authors also pointed out that dietary Se affected both the composition of existing microbiota and the establishment of gastrointestinal microflora (Kasaikina et al., 2011). In the recent experiments in rats, it was shown that high doses of Se (as selenite) partially restored the ranks of phylum and genus of the gut flora after the exposure to methylmercury. The authors also pointed out that the host’s Se level was related to the state of the gut microbiome (Liu et al., 2019). Gangadoo et al. (2019) reported that exposure to Se nanoparticles affected the diversity and structure of chicken caecal microbiota *in vitro*. To conclude, it is tempting to speculate that the alterations of the host organism due to Se dietary levels (Kipp et al., 2009, 2012; Peters et al., 2018; Zhai et al., 2019) may be partially related to the gut microbiota. However, further studies should support such a hypothesis.

Another important aspect of the gut microbiota in line with the scope of the current review is related to its role in maintaining BBB integrity. Pathological alterations in gut microbiota induce the increased production of toxic metabolites and reduced production of beneficial compounds like short-chain fatty acids. The metabolic change affects the balance of pro-inflammatory and anti-inflammatory cytokines and other immune factors, promoting the decline of the gut epithelial barrier. This results in concomitant activation of local and distant immune cells and dysregulation of enteric neurons and glia (Welcome, 2019). Gut flora also appears to have a role in the induction of BBB properties; in the absence of normal gut microbiota in the mouse dams, the expression of BEC claudin-5 and occludin is diminished and an increase in BBB permeability is observed in the offspring (Braniste et al., 2014). Notably, more research is currently required to shed light on the exact molecular switches that control the processes in the histohematic barriers of the gut and brain (Welcome, 2019). Even less information exists regarding the role of Se in these processes. There was a report that Se uptake (as selenite) to the brain increased in lipopolysaccharide treated female mice, whereas in males, no increased BBB permeability for selenite was observed under such conditions (Minami et al., 2002). The sex-specific phenotype in Se metabolism is rather well-described – the reader is referred to the review by Seale et al. (2018b). Additionally, Se treatment (100 nmol/L) was shown to inhibit glucose-induced expression of adhesion molecules in the human umbilical vein endothelial cells (Zheng et al., 2008); however, the research in brain endothelium is still required to evaluate the role of Se in cell adhesion in BBB and BCB. Oztas et al. (2007) reported that sodium selenite (4 ppm in rat dams drinking water) and vitamin E supplementation had a beneficial effect on BBB integrity in the rat pups. However, the studies on the effect of dietary Se and/or selenoprotein expression on, e.g., tight junction protein expression or systemic *in vivo* research on Se/selenoprotein in BBB and BCB permeability are currently absent, to the best of the author’s knowledge.

## Conclusion and Perspectives

The understanding of brain Se transport has considerably improved, first of all, over the past two decades. The primary SELENOP/LRP8-dependent mechanism of Se entering the brain is rather well described. However, even in this respect, there are still some gaps remaining. For instance, ApoE, sharing the brain uptake receptor with SELENOP, appears to be important for the regulation of tight junction integrity at the BBB (Blanchette and Daneman, 2015). This is tempting to speculate on possible interplay, but such speculations require a further warrant, especially *in vivo* research.

Another currently understudied and important aspect of Se interplay with BBB and BCB may be accomplished through immune and inflammatory pathways. Individual selenoproteins are known to be involved in regulating inflammation and immunity. Se deficiency negatively impacts immune cells during activation, differentiation, and proliferation through redox signaling, oxidative burst, calcium flux, and the subsequent effector functions of immune cells (Huang et al., 2012; Avery and Hoffmann, 2018; Toledo et al., 2020). For instance, the potential inhibition of the nuclear factor kappa-B (NFκB) signaling pathway by Se and selenocompounds is often considered (Santamaría et al., 2005; Vunta et al., 2007; Duntas, 2009; Gholami et al., 2015). Importantly, Dreher et al. (1997) demonstrated *in vitro* that human SELENOP gene’s promoter was cytokine responsive. Consequently, inflammatory processes may affect SELENOP production by the liver thus influencing brain Se uptake.

Se metabolism is known to be affected by sex (Schomburg, 2016; Seale et al., 2018b). On the other hand, common neurological diseases, e.g., Alzheimer’s disease have pronounced both marked sex-dependency (Ferretti et al., 2018) and involvement in the BBB decline (Zenaro et al., 2017). Additionally, studies in mice have shown higher liver and kidney expression of *Selenop* in females than that in males (Schomburg et al., 2007), so SELENOP/LRP8-brain Se uptake pathway may also exert sex-dependence. Thus, another aspect for future research of Se at the brain barriers may be related to improving our understanding on sex-related differences, including the effects of dietary Se, selenoproteins, and selenometabolites on permeability and function of BBB, BCB as well as gut-brain axis and brain immunity. The same corresponds to the effect of age and aging on the brain barrier functions and the related effects of Se and selenoproteins (Huang et al., 2012).

Many studies indicate the critical importance of the exact chemical speciation of essential trace elements (Templeton et al., 2000) in determining their biological activity (Michalke et al., 2018). For instance, in *post mortem* studies in the human brain from Alzheimer’s disease patients, it was demonstrated that the Se distribution pattern in the brain is seriously distorted (Bellinger et al., 2008; Rueli et al., 2015). SELENOP was shown to be co-localized with Alzheimer’s disease brain tissue lesions – Aβ plaques and neurofibrillary tangles (Bellinger et al., 2008). Moreover, in a further study of the same group, the increased release of SELENOP from the *choroid plexus* to the CSF in Alzheimer’s disease patients was reported (Rueli et al., 2015). Speciation studies in CSF demonstrated that exposure of the brain tissue to hexavalent Se may be involved in Alzheimer’s (Vinceti et al., 2017a, 2019) or amyotrophic lateral sclerosis pathology (Vinceti et al., 2013b; Mandrioli et al., 2017; Violi et al., 2020). Unfortunately, the exact molecular pathways of Se in the neurodegenerative processes, including the transport through the neural barrier endothelia have only been studied scarcely. The diverse biological activities of Se urgently require systematic studies concerning its behavior at the BBB and BCB and its role in maintaining barrier function and integrity. The investigations on *in vitro* BBB or BCB models, analogous to those published by Bornhorst et al. (2012) and Müller et al. (2018) for other elements could help to further clarify barrier processes regarding Se and seleno-species. Mapping of brain-barrier regions with laser ablation inductively coupled plasma mass spectrometry (LA-ICP-MS) technology for Se in combination with microscopy and histology is encouraged to support this interesting research field. Recent technological advances in analytical science are now enabling the study of Se transport, its spatial and chemical distribution at an unprecedented level of detail. Finally, the question of Se efflux from the brain is not properly addressed. It should be noted that uptake of Se into the brain compartment without a balanced Se-discharge mechanism could finally lead to local, brain-compartment related Se-overexposure. In contrast to this necessary balance, there is a considerable misbalance between the studies concerning Se entering the brain or particular brain cells and these on Se leaving the CNS. Along with thorough literature research on studies about Se-efflux from brain compartment no references were found. Although similar mechanisms may be involved in Se discharge from the brain, i.e., involving SELENOP and minor low molecular weight Se-metabolites, there are no relevant studies supporting this notion, to the best of the authors’ knowledge. That misbalance should be addressed in future research.

## Author Contributions

NS and BM conceived the agenda for the review. NS contributed to all sections. ED largely contributed to section “Blood–Brain Barrier, Blood–Cerebrospinal Fluid Barrier, and Neurovascular Unit” and prepared all the figures. BB mainly contributed to sections “Introduction,” “Blood–Brain Barrier, Blood–Cerebrospinal Fluid Barrier, and Neurovascular Unit,” and “Selenium Transport to The Brain – Selenoprotein P and Low Molecular Weight Selenium-Species.” BM mainly contributed to sections “Introduction,” “Selenium Transport to The Brain – Selenoprotein P and Low Molecular Weight Selenium-Species,” and “Conclusion and Perspectives.” All authors contributed to the editing and discussion and agreed to submit the manuscript in its current state.

## Conflict of Interest

The authors declare that the research was conducted in the absence of any commercial or financial relationships that could be construed as a potential conflict of interest.
